# Global Trends in Social Prescribing: Web-Based Crawling Approach

**DOI:** 10.2196/46537

**Published:** 2023-05-22

**Authors:** Hocheol Lee, Sang Baek Koh, Heui Sug Jo, Tae Ho Lee, Hae Kweun Nam, Bo Zhao, Subeen Lim, Joo Aeh Lim, Ho Hee Lee, Yu Seong Hwang, Dong Hyun Kim, Eun Woo Nam

**Affiliations:** 1 Department of Health Administration Software Digital Healthcare Convergence College Yonsei University Wonju Republic of Korea; 2 Yonsei Global Health Center Yonsei University Wonju Republic of Korea; 3 Department of Preventive Medicine Wonju College of Medicine Yonsei University Wonju Republic of Korea; 4 Department of Health Policy and Management Kangwon National University School of Medicine Chuncheon Republic of Korea; 5 Korea Industry Development Institute Gangneung Republic of Korea; 6 Department of Information Statistics Yonsei University Wonju Republic of Korea

**Keywords:** social prescribing, social loneliness, National Health Service, aging population, web crawling, sustainable model, Google Trend, web-based data, NPI, health care model, primary care, digital health intervention, implementation, health care professional

## Abstract

**Background:**

Social loneliness is a prevalent issue in industrialized countries that can lead to adverse health outcomes, including a 26% increased risk of premature mortality, coronary heart disease, stroke, depression, cognitive impairment, and Alzheimer disease. The United Kingdom has implemented a strategy to address loneliness, including social prescribing—a health care model where physicians prescribe nonpharmacological interventions to tackle social loneliness. However, there is a need for evidence-based plans for global social prescribing dissemination.

**Objective:**

This study aims to identify global trends in social prescribing from 2018. To this end, we intend to collect and analyze words related to social prescribing worldwide and evaluate various trends of related words by classifying the core areas of social prescribing.

**Methods:**

Google’s searchable data were collected to analyze web-based data related to social prescribing. With the help of web crawling, 3796 news items were collected for the 5-year period from 2018 to 2022. Key topics were selected to identify keywords for each major topic related to social prescribing. The topics were grouped into 4 categories, namely Healthy, Program, Governance, and Target, and keywords for each topic were selected thereafter. Text mining was used to determine the importance of words collected from new data.

**Results:**

Word clouds were generated for words related to social prescribing, which collected 3796 words from Google News databases, including 128 in 2018, 432 in 2019, 566 in 2020, 748 in 2021, and 1922 in 2022, increasing nearly 15-fold between 2018 and 2022 (5 years). Words such as health, prescribing, and GPs (general practitioners) were the highest in terms of frequency in the list for all the years. Between 2020 and 2021, COVID, gardening, and UK were found to be highly related words. In 2022, NHS (National Health Service) and UK ranked high. This dissertation examines social prescribing–related term frequency and classification (2018-2022) in Healthy, Program, Governance, and Target categories. Key findings include increased “Healthy” terms from 2020, “gardening” prominence in “Program,” “community” growth across categories, and “Target” term spikes in 2021.

**Conclusions:**

This study’s discussion highlights four key aspects: (1) the “Healthy” category trends emphasize mental health, cancer, and sleep; (2) the “Program” category prioritizes gardening, community, home-schooling, and digital initiatives; (3) “Governance” underscores the significance of community resources in social prescribing implementation; and (4) “Target” focuses on 4 main groups: individuals with long-term conditions, low-level mental health issues, social isolation, or complex social needs impacting well-being. Social prescribing is gaining global acceptance and is becoming a global national policy, as the world is witnessing a sharp rise in the aging population, noncontagious diseases, and mental health problems. A successful and sustainable model of social prescribing can be achieved by introducing social prescribing schemes based on the understanding of roles and the impact of multisectoral partnerships.

## Introduction

### Background

Nearly one-third of the population in industrialized countries is affected by social loneliness, with 1 in every 12 people presenting severe symptoms. Studies show that this condition increases the risk of premature mortality by 26% [1]. Persistent social loneliness closely relates to adverse health outcomes such as coronary heart disease, stroke, depression, cognitive impairment, and Alzheimer disease, all of which pose a risk as great as obesity or smoking [2-4]. Social loneliness occurs due to the lack of contact with people [5].

In October 2018, the UK government announced an approach to deal with the negative impact of loneliness, namely *A Connected Society: A Strategy for Tackling Loneliness*, a strategy to connect community resources to address social loneliness at the state level [6]. Accordingly, the government appointed a Minister for Loneliness and implemented social prescribing services, which are nonmedical interventions based on local communities, to tackle social loneliness.

Social prescribing is a health care model wherein general practitioners (GPs), engaged in providing primary health care, prescribe nonpharmacological interventions in partnership with local communities [7]. Users of this service are involved in community activities for 12 weeks on average, including gardening, art therapy, music therapy, sports classes, reading, walking, and volunteering [8]. The UK government allocates link workers to help users access social prescribing initiatives easily.

Social prescribing interventions are available nationwide in the United Kingdom with financial support based on the long-term plan of the National Health Service (NHS) [9]. The government announced the *NHS Five Year Forward View* and *the NHS Long-Term Plan* [9,10], defining social prescribing as a “comprehensive model of personalized care” for planning and implementing social prescribing interventions involving 900,000 persons by 2023 [11].

Instituted in the United Kingdom in 2018, the concept of social prescribing is being implemented worldwide in 2023, especially in Korea [12], the United States [13], Australia [14], Singapore [15], and New Zealand [16]. Social prescribing pilot projects are currently being implemented globally in some communities, facilities, and regions. A growing number of studies are assessing the effectiveness of social prescribing initiatives and programs to formulate them into policies [12-16].

As social prescribing interventions are expanding globally, it is becoming increasingly important to prove their effectiveness. The British Department of Health and Social Care established the National Academy of Social Prescribing to create the necessary framework to promote and implement the program by building an evidence base for investigating and evaluating the effect of social prescribing [11]. Despite these efforts, many health care professionals and policy makers have stressed a lack of strong evidence regarding the effectiveness of social prescribing programs [17-20]. They indicated the difficulty in assessing social prescribing schemes objectively due to the brief period of their application and the lack of control groups and related research studies [21]. Therefore, it is necessary to prepare evidence-based plans considering various global trends and research findings to disseminate social prescribing worldwide, centering on the United Kingdom.

### Aim

This study aims to identify global trends in social prescribing over the past 5 years (ie, since its introduction in 2018). To this end, we intend to collect and analyze words related to social prescribing worldwide and evaluate various trends of related words by classifying the core areas of social prescribing. Based on these findings, we propose to provide evidence for implementing social prescribing.

## Methods

### Study Design

This cross-sectional study analyzed data posted between January 1, 2018, and December 31, 2022, to understand global trends in social prescribing programs.

### Data Collection

To analyze web-based data related to social prescribing, we collected Google’s searchable data. Google’s database was selected as it is the most widely used search engine and has the largest uploaded posts. To secure data objectivity, newspapers were primarily collected (crawling). As of December 31, 2022, there were approximately 6,720,000 searches for “Social Prescribing” in the Google News database. For web crawling, the Beautiful Soup module (developed in Python; version 3.7; Python Software Foundation) was used, and Chrome driver and *Selenium* packages were used to collect data from the Google News database. In the web crawling process, “Social Prescribing” was selected as the target word.

With the help of web crawling, a total of 3796 news items were collected, including 128 in 2018, 432 in 2019, 566 in 2020, 748 in 2021, and 1922 in 2022. Data were classified using morphology analysis, and among the classified words, unused articles and punctuation marks (“.,” “…,” etc) were removed to select 26,897 final words. Morphology analysis is a natural language processing technique that focuses on analyzing the structure and formation of words and breaking them down into their constituent parts, such as roots, prefixes, and suffixes. By understanding these components and their relationships within the collected data, we can gain better insights into the context and meaning of the content. This method allows us to effectively filter and organize the data obtained from web crawling, identifying relevant terms, phrases, and patterns related to the subject of social prescribing. By implementing morphology analysis, we can enhance the accuracy and reliability of our research findings, providing a more comprehensive understanding of the trends and developments in social prescribing across various news sources.

### Word Selection

Three health experts held discussions to identify words related to social prescribing from the final 26,897 words selected using web crawling. Among the 3 health experts, 1 is a professor at a university with a doctorate in public health, and the other 2 members are link workers in a Korean pilot program for social prescriptions.

They selected key topics to identify keywords for each major topic related to social prescribing, and a toolkit on how to implement social prescribing, published by the World Health Organization (WHO), was referred in the selection process. Accordingly, the topics were grouped into 4 categories, namely Healthy, Program, Governance, and Target, and keywords were selected for each topic.

### Statistical Analysis

Text mining was used to determine the importance of words collected from new data. We have constantly raised the difficulty of analysis due to meaningless stop words, such as articles and prepositions (a, the, to, in, etc). The *Natural Language Toolkit* package was preferred for analyzing selected words, and frequency analysis was used to understand the frequency of word occurrence. Frequency analysis was considered as a suitable analysis due to advantages such as its speed of operation, easy access, factual verification of data, and easy recognition of English words. The selected data were analyzed as follows: first, a word cloud was visualized from 2018 to 2022 to identify the keywords for each year; second, topic-specific words were categorized into Healthy, Program, Governance, and Target and visualized as a word cloud; and third, the frequency of occurrence of words between 2018 and 2022, based on topics such as Healthy, Program, Governance, and Target, was represented in a line graph.

### Ethical Considerations

The authors did not seek institutional review board approval as this was not human-subject research. All of the websites observed, and their archived versions were publicly available.

## Results

### Crawling Data

**Figure 1:**
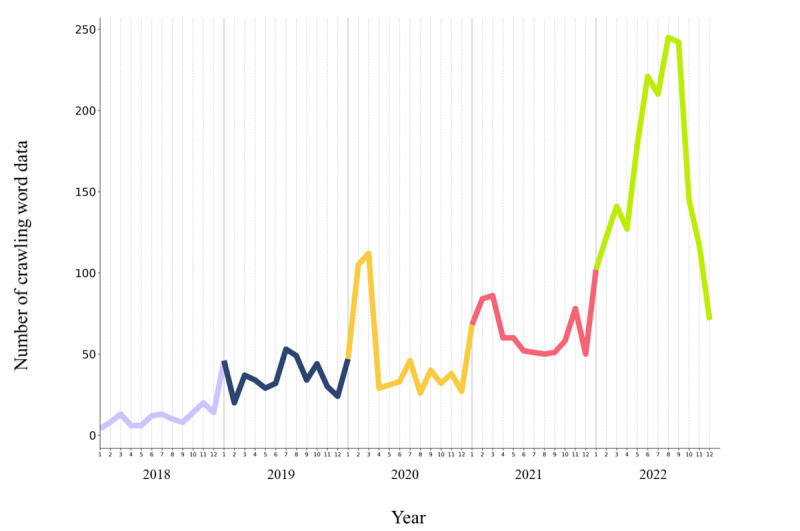
Number of crawling word data from 2018 to 2023.

Web crawling was used to collect 3796 words related to social prescribing, including 128 in 2018, 432 in 2019, 566 in 2020, 748 in 2021, and 1922 in 2022, increasing nearly 15-fold between 2018 and 2022 (Table 1). The line graph in Figure 1 shows a steady increase in the number of words related to social prescribing from January 2018 to December 2020 and a rapid increase in the first half of 2020 and 2022, respectively (Figure 1).

**Table 1:** Number of crawl data.

	2018, n (%)	2019, n (%)	2020, n (%)	2021, n (%)	2022, n (%)
January	4 (3.1)	46 (10.6)	47 (8.3)	68 (9.1)	102 (5.3)
February	8 (6.3)	20 (4.6)	105 (18.6)	84 (11.2)	122 (6.3)
March	13 (10.2)	37 (8.6)	112 (19.8)	86 (11.5)	141 (7.3)
April	6 (4.7)	34 (7.9)	29 (5.1)	60 (8.0)	127 (6.6)
May	6 (4.7)	29 (6.7)	31 (5.5)	60 (8.0)	179 (9.3)
June	12 (9.4)	32 (7.4)	33 (5.8)	52 (7.0)	221 (11.5)
July	13 (10.2)	53 (12.3)	46 (8.1)	51 (6.8)	210 (10.9)
August	10 (7.8)	49 (11.3)	26 (4.6)	50 (6.7)	245 (12.7)
September	8 (6.3)	34 (7.9)	40 (7.1)	51 (6.8)	242 (12.6)
October	14 (10.9)	44 (10.2)	32 (5.7)	58 (7.8)	145 (7.5)
November	20 (15.6)	30 (6.9)	38 (6.7)	78 (10.4)	117 (6.1)
December	14 (10.9)	24 (5.6)	27 (4.8)	50 (3.7)	71 (3.7)
Total	128 (100.0)	432 (100.0)	566 (100.0)	748 (100.0)	1922 (100.0)

### Word Cloud

**Figure 2:**
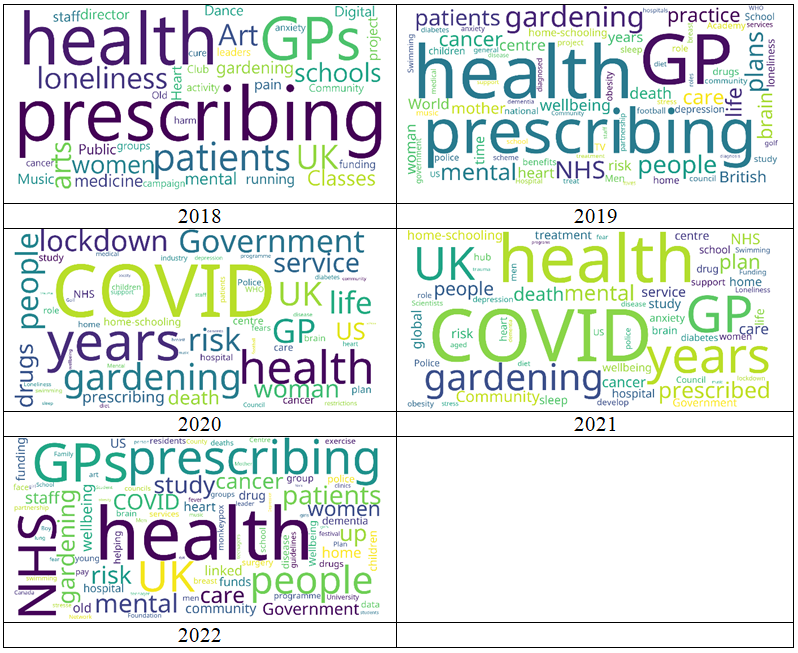
Social prescribing word cloud by year. GP: general practitioner; NHS: National Health Service.

We generated word clouds for words related to social prescribing collected from news between 2018 and 2022 (Figure 2). Overall, words such as health, prescribing, and GPs were highest in terms of frequency in the list for all the years. Between 2020 and 2021, COVID, gardening, and UK were found to be highly related words. In 2022, NHS and UK ranked high.

**Figure 3:**
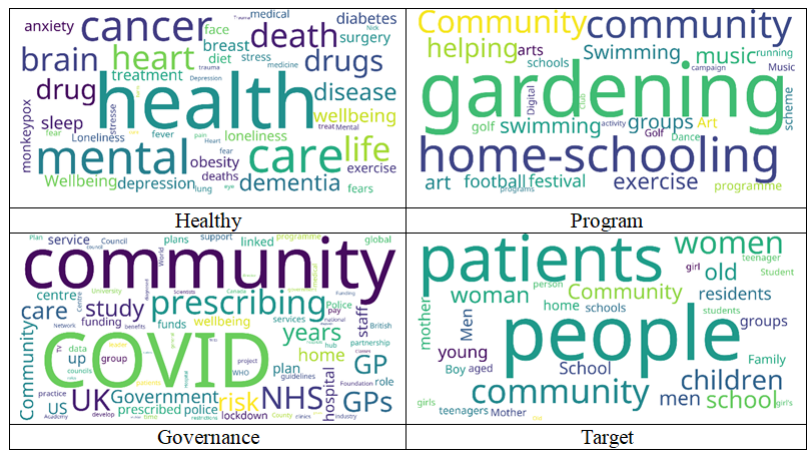
Social prescribing word cloud by key category. GP: general practitioner; NHS: National Health Service.

Crawling data were classified based on the core keywords of social prescribing from 2018 to 2022, and word clouds were generated (Figure 2). Under the keyword “Healthy,” health was the most frequently used word, followed by mental, care, and cancer. The order of frequency under “Program” was gardening, home-schooling, and community. Under “Governance,” it was COVID, community, and NHS; the most frequently occurring words under “Target” were patients, people, community, women, and old (Figure 3).

Under “Healthy,” words such as mental, care, cancer, and sleep were found to increase sharply from 2020 onward, whereas “death” increased until 2021 and decreased in 2022.

In the “Program” category, “gardening” was the most frequent used word from 2018 to 2022, and it increased continuously. Other words such as community, home-schooling, digital, swimming, and music had increased continuously, since 2018. In particular, “community” showed a rapid increase from 2020 onward.

In the “Governance” category, words such as community, GPs, NHS, and prescribing increased rapidly from 2021 onward. Moreover, words such as risk, study, government, and home also increased in 2021.

In the “Target” category, words such as people, patient, women, aged, and children increased sharply from 2021, and “community” increased steadily from 2018 onward.

### Trend Changes in Social Prescribing by Key Category

**Figure 4:**
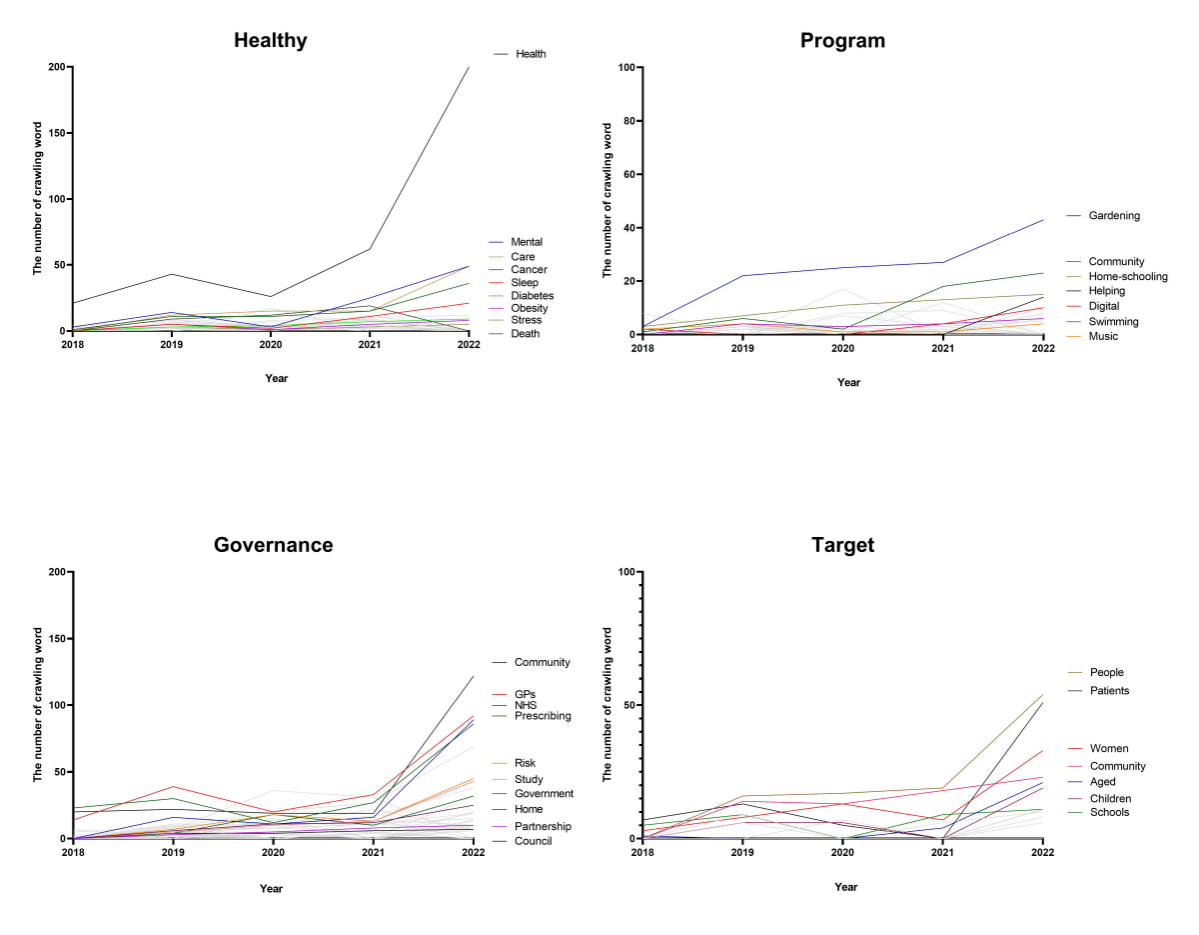
Trend changes in social prescribing by key category. GP: general practitioner; NHS: National Health Service.

Words related to social prescribing were classified into categories such as Healthy, Program, Governance, and Target, and changes in frequency between 2018 and 2022 were examined and visualized in a line graph (Figure 4).

## Discussion

### Principal Findings

The purpose of this study is to identify global trends in social prescribing over a 5-year period from 2018 to 2022 and analyze the related words by applying the identified key topics to provide evidence for implementing social prescribing. The number of new papers related to social prescribing increased 15-fold from 128 in 2018 to 1922 in 2022, and the rise was particularly sharp in 2022. The main reason for this increase in the United Kingdom was the nationwide implementation of social prescribing programs. Among the major concerns, as part of the social prescribing movement, the UK government announced a plan in August 2022 to provide walking and cycling through GPs, and 11 local authorities planned to invest US $15.9 million in pilot programs [22,23]. As social prescribing services were used for daily health care activities using community resources, there has been growing interest among institutions, facilities, and media houses, including British citizens. Alongside the UK Ministry of Health’s active support for social prescribing, various institutions have also adopted social prescribing programs [24].

The global spread of social prescribing is evidenced in all the policies implemented at the national level in many countries, other than news reports. Introduced in the United Kingdom, other countries such as the United States, China, Korea, Germany, Denmark, Austria, Finland, Sweden, Spain, Singapore, Ireland, New Zealand, Portugal, Canada, and the Netherlands have launched social prescribing pilots and policy reviews [25-34].

This study extracted keywords from news items using web crawling and divided them into 4 categories, in reference to the WHO’s social prescribing guidelines, to identify international trends from 2018 to 2022. The study results are as follows. First, in the “Healthy” category, the top 3 words by year are mental, cancer, and sleep. The UK NHS was established for the purpose of addressing mental health problems, such as social isolation and depression, using community resources through social prescribing, and appropriate implementation is evidenced by the continuous increase in the frequency of the word “mental” [35]. The UK NHS provides social prescribing through community GPs to patients diagnosed with cancer. Nearly 70% of patients with cancer develop mental and physical health problems, heart diseases, shortness of breath, nausea, and hair loss. To help them deal with these problems, social prescribing services are provided using local community resources through the digital platform, such as Element [36]. According to the WHO guidelines, social prescribing is recommended for people experiencing sleep disturbances, leading to its high frequency while referencing [37].

Second, under the category of “Program,” gardening was the most frequently used word, followed by community, home-schooling, and digital. Gardening is a representative initiative recommended by the UK NHS under the concept “green social prescription” to promote mental health and develop confidence among service users through gardening activities [38,39]. Gardening is a nonpharmacological intervention that fosters mental health, alleviates stress and loneliness as well as provides participants economic benefit worth US $1037.5 per year [40].

Third, since social prescribing programs are implemented using local community resources, “Governance” is recommended as a key category. The most important keyword was “community.” This word is increasingly mentioned, as all programs and schemes of social prescribing are conducted using local community resources, including human resources, venues, funding, and goods. In this context, the NHS has been nurturing the option of professional link workers and a plan to expand staffing [41]. According to research on social prescribing at the community level, the success of social prescribing is closely related to the organic connection of community resources and trust within the community. In particular, individuals, community infrastructure, resources, and personnel need to be connected based on trust relationships, and this role should be performed by link workers (coordinators) who have received specialized training [42].

Lastly, regarding the “Target” of social prescribing, the UK NHS recommends four main targets of social prescribing: (1) people with one or more long-term conditions; (2) those with low-level mental health issues and require support; (3) lonely or isolated people; and (4) people with complex social needs that affect their well-being. Based on this, the targets are becoming more specific and are expanding every year to include elderly patients with cancer, mentally ill patients, substance addicts, and patients with chronic diseases [43]. This study also found that the relationship between the targets of all groups, such as men, women, children, and the elderly, is rapidly increasing, indicating that social prescribing services are being provided to local residents.

As social prescribing is rapidly spreading worldwide, experts are beginning to agree on the need for such programs; however, most of them argue that there are many problems to be addressed due to the complexity of linking multiple sectors during implementation.

First, experts indicate lack of research as a major concern. In this study, “study” was one of the main related words. To prescribe social prescribing as a national policy worldwide, it is necessary to establish evidence-based plans. However, few studies provide evidence of social prescribing programs being conducted in other countries, except the United Kingdom. For promoting a social prescribing system, case studies that are relevant to various fields are required, including manpower, funding, delivery system, community volunteers, and linkage with a national insurance policy [44].

Second, monitoring and risk management are essential for the successful implementation of social prescribing programs through governance based on community partnerships [45]. This study also observed a continuous increase in the frequency of “risk” as a related word. An efficient method of risk management is to train link workers in monitoring and managing risk to establish a comprehensive monitoring system.

Third, due to a lack of dedicated management departments, the social prescribing system connects various stakeholder institutions that are centered on the NHS, such as medical facilities, private service providers, platform development companies, and research institutes. The network of stakeholders, related to social prescribing, is rapidly growing, thus, making it difficult to be managed by a single team of the NHS. It is recommended that at least one public institution dedicated to managing social prescribing should be established.

However, there are limitations to this study that need to be acknowledged—first, web crawling is based on data sourced from the Google News database. Although this is to secure data objectivity, citizens’ opinions and research results are not reflected. Second, data were collected only from English newspapers. Third, this study was unable to conduct analysis between regions and countries, as it comprehensively collected and analyzed news data from around the world. In future research, it will be necessary to investigate the trends of social prescriptions between regions and countries. As this study could not fully reflect the global spread of social prescribing, follow-up studies should design a more sophisticated crawling technique to collect data in other languages and English.

### Conclusions

Based on this study, it is clear that the concept of social prescribing is gaining global acceptance and is becoming a national policy worldwide. Needless to say, social prescribing is gaining prominence as the world is witnessing a sharp increase in the aging population, noncontagious diseases, and mental health problems. However, it is necessary to provide sufficient evidence for implementing social prescribing programs worldwide. A successful and sustainable model of social prescribing can be achieved only by introducing social prescribing schemes based on the understanding of roles and the impact of multisectoral partnerships.

## Abbreviations

GP: general practitioner
NHS: National Health Service
WHO: World Health Organization

## Data Availability

The data sets generated and analyzed during this study are available from the corresponding author upon reasonable request.
